# Application of Gd-EOB-DTPA-enhanced magnetic resonance imaging (MRI) in hepatocellular carcinoma

**DOI:** 10.1186/s12957-020-01996-4

**Published:** 2020-08-22

**Authors:** Xue-Qin Li, Xing Wang, Da-Wei Zhao, Jun Sun, Jiao-Jiao Liu, Dong-Dong Lin, Guang Yang, Hui Liu, Zhen-Ying Xia, Cui-Yu Jia, Hong-Jun Li

**Affiliations:** 1 Department of Radiology, Beijing YouAn Hospital, Capital Medical University, Beijing, 100069 China; 2 Department of Surgery, Beijing YouAn Hospital, Capital Medical University, Beijing, 100069 China; 3 Department of Pathology,Beijing YouAn Hospital, Capital Medical University, Beijing, 100069 China

**Keywords:** Magnetic resonance imaging, Gd-EOB-DTPA, Hepatocellular carcinoma

## Abstract

**Background:**

Hepatocellular carcinoma (HCC) is the most common malignant tumor of the liver, and its morbidity and mortality have been increasing in recent years. The early diagnosis and prompt treatment of small HCC are crucial to improve the prognosis and quality of life of patients. In China, hepatitis B virus infection is the main cause. HCC with a single tumor nodule of ≤ 3 cm in diameter, or HCC with a number of nodules, in which each nodule is ≤ 2 cm in diameter, with a total diameter of ≤ 3 cm, is considered as small HCC. The MRI liver-specific contrast agent can detect small HCC at the early stage. This has important clinical implications for improving the survival rate of patients.

**Main body:**

Gd-EOB-DTPA-enhanced MRI can significantly improve the sensitivity and specificity of the detection of HBV-related small hepatocellular carcinoma, providing an important basis for the clinical selection of appropriate personalized treatment. Gd-EOB-DTPA-enhanced MRI can reflect the degree of HCC differentiation, and the evaluation of HCC on Gd-EOB-DTPA-enhanced MRI would be helpful for the selection of the treatment and prognosis of HCC patients. The present study reviews the progress of the application of Gd-EOB-DTPA in the early diagnosis of small HCC, its clinical treatment, the prediction of the degree of differentiation, and the assessment of recurrence and prognosis of HCC, including the pharmacoeconomics and application limitations of Gd-EOB-DTPA. The value of the application of HCC with the Gd-EOB-DTPA was summarized to provide information for improving the quality of life and prolonging the survival of patients.

**Conclusion:**

Gd-EOB-DTPA-enhanced MRI has the diagnostic capability for small HCC with a diameter of ≤ 2 cm. This will have a broader application prospect in the early diagnosis of small liver cancer with a diameter of ≤ 1 cm in the future. The relationship between GD-EOB-DTPA-MRI and the degree of HCC differentiation has a large research space, and Gd-EOB-DTPA is expected to become a potential tool for the preoperative prediction and postoperative evaluation of HCC, which would be beneficial for more appropriate treatments for HCC patients.

## Background

Hepatocellular carcinoma (HCC) is the most common primary malignant tumor of the liver, the sixth most common cancer in the world, and the second largest cause of cancer-related death [1, 2]. Worldwide, China, South Korea, and sub-Saharan Africa have the highest incidence of HCC [1], and Chinese patients account for more than 50% of the total number of HCC cases and deaths [3]. In China, hepatitis B virus (HBV) infection is the main cause of HCC, and these patients present with HBV-associated hepatocellular carcinoma. HCC has an insidious onset and has no obvious symptoms in the early stage, with a high rate of missed diagnosis and misdiagnosis. When the final diagnosis is made with typical symptoms, the stage of the disease is often advanced [3]. With the exception of some patients who received early diagnosis and prompt surgical resection or liver transplantation, the overall prognosis of HCC remains poor [4], with a median survival time of < year and a 5-year survival rate of < 10% [5], and a high recurrence rate [6]. HCC with a single tumor of ≤ 3.0 cm is usually referred to as small HCC [7]. In China, the standards for small HCC formulated by the Chinese Liver Cancer Pathology Collaboration Group is adopted. That is, HCC with a diameter of a single tumor nodule of ≤ 3 cm, or HCC with a number of nodules, in which each nodule is ≤ 2 in diameter, with a total diameter of ≤ 3 cm, is considered as small HCC. Some studies have revealed that different assessment methods for small HCC lesions with a diameter of < 2.0 cm can affect the preoperative Barcelona Clinic Liver Cancer (BCLC) staging classification in some patients [8], thereby influencing the selection of treatment options. Therefore, the early diagnosis of small HCC is crucial for patients in choosing the appropriate treatment at the right time.

Unlike most solid cancers, HCC can be diagnosed with non-histological evidence [9]. The advances in computed tomography (CT), magnetic resonance imaging (MRI) and other imaging technologies have improved the methods for the diagnosis and evaluation of HCC [10–12]. During the early HCC screening, CT or MRI examination is required for patients with alpha fetoprotein (AFP) > 100 ng/ml or ultrasound examination results of nodules with a diameter of > 1.0 cm [1]. Compared with the conventional contrast-enhanced CT, MRI provides high sensitivity and spatial resolution, with the advantages of increased soft tissue contrast and no radiation damage [13]. Meanwhile, the information regarding the patient’s metabolism, physiology, and pathology can be analyzed, which could be valuable in making treatment decisions. Therefore, MRI has become one of the most commonly used methods in clinical liver examinations. The feasibility of conventional enhanced MRI in the diagnosis of HCC has been widely recognized [14]. However, there is still a lack of accuracy and sensitivity in the diagnosis of small HCC lesions with a diameter ≤ 2.0 cm [8, 15]. Gadolinium ethoxybenzyldiethylenetriaminepentaacetic acid (Gd-EOB-DTPA) is the liver-specific contrast enhancement agent presently used for the diagnosis of HCC. MRI with Gd-EOB-DTPA enhancement is superior, when compared to enhanced CT and conventional contrast-enhanced MRI, in the diagnosis of small liver lesions, and the differentiation of benign and malignant nodules [14, 16–18]. The present study summarizes the research progress of Gd-EOB-DTPA-enhanced MRI in the early diagnosis of small HCC and the assessment of HCC, in an attempt to provide reference for its clinical application.

## Main text

### Basic principle and mechanism of Gd-EOB-DTPA

Gd-EOB-DTPA is a liver- and bile-specific MRI contrast agent, which is formed by adding fat-soluble ethoxybenzyl (EOB) to the molecular structure of Gd-EOB-DTPA. In addition to the dynamic enhancement features of traditional MRI contrast agents, Gd-EOB-DTPA also has unique biological characteristics [6]. On the one hand, the multi-phase dynamic enhancement effect similar to Gd-DTPA can be obtained by shortening the T1 relaxation time of tissues [8]. On the other hand, after the intravenous injection of Gd-EOB-DTPA, 50% of the contrast would pass through the organic anion transporting polypeptide 1B3 (OATP1B3) that exists on the liver cell membrane of the liver blood sinus, which would in turn be absorbed by normal liver cells after 10–20 min. Then, this is excreted into the biliary tract through multidrug resistance-associated protein 2 (MRP2) on the biliary tract of the liver membrane. The period of this phase is called the hepatobiliary specific period or hepatobiliary phase [19, 20]. The remaining contrast agent, similar to Gd-DTPA, can be excreted through the kidney. This dual clearance pathway can compensate for each other when liver or kidney function is damaged, thereby ensuring higher safety [21]. In the hepatobiliary specific period images, normal liver cells continue to ingest when Gd-EOB-DTPA passes through the liver, which shortens the T1 relaxation time of normal liver tissues, and shows the hyperintensity signal on T1WI. On the contrary, HCC cells cannot specifically ingest Gd-EOB-DTPA due to the reduced OATP1B3 expression level, showing a relative hypointensity [22]. The contrast between the surrounding isosignal intensity of the cirrhotic nodules with hyperintense normal hepatic tissues makes the lesion clearer and easier to diagnose. The uptake of intrahepatic Gd-EOB-DTPA is considered as an indicator of liver cell function [23]. The chemical structure of Gd-EOB-DTPA remains unchanged during the metabolic cycle and is completely cleared from plasma within 24 h after injection. Based on the above characteristics, the MRI of the hepatobiliary specific contrast agent Gd-EOB-DTPA can not only evaluate the blood supply pattern of HCC lesions by utilizing dynamic enhanced scanning, but also evaluates the function of liver cells by evaluating the signal changes of lesions on the hepatobiliary phase [23].

Most small HCC lesions show a typical characteristic multiphase dynamic enhancement, which is characterized by “fast in and wash out”. This is called rich blood supply of small HCC. Furthermore, at the liver-gallbladder specific stage, this shows a low signal [9], which enables the diagnosis to be easy. However, when there is a lack of blood supply, atypical manifestations and intrahepatic focal perfusion abnormalities can occur, causing the conventional qualitative diagnosis of enhanced MRI to be often difficult. The Liver Imaging Report and Data System (LI-RADS) 2018 defined hepatic lesions with an arterial enhancement lower than or equal to the normal liver parenchyma as hepatic hypovascular lesions [24], which is relatively difficult to diagnose before surgery. Under this condition, when Gd-EOB-DTPA is used to enhance MRI, this would be more helpful for the qualitative diagnosis and detection of small HCC. The prominent advantage of Gd-EOB-DTPA-enhanced MRI lies in the hepatobiliary phase. Most HCC presents with a relatively low signal due to the low uptake caused by the abnormal content of hepatocytes, and the strong contrast with the surrounding liver parenchyma, which greatly improves the detection rate of lesions with insufficient blood supply [25].

### Clinical application of Gd-EOB-DTPA

#### The early diagnosis of HBV-associated small HCC

In recent years, with the in-depth research on hepatocyte-specific contrast agents at home and abroad, some literatures reported that compared with dynamic enhancement of multislice spiral CT, plain MRI, and conventional extracellular space contrast agent Gd-DTPA, Gd-EOB-DTPA-enhanced MRI for the accuracy of diagnosis of early liver cancer has been proven to be more advantageous [11, 14]. Europe, the USA, and Japan in 2004 and 2008 respectively assessed liver tumors using Gd-EOB-DTPA-enhanced MRI, and China has also started its application [23] in 2010, which has become widely used in clinical practice. This can significantly improve the detection sensitivity and specificity for HBV-related small liver cancer, and its clinical value has been confirmed.

The enhanced MRI of Gd-EOB-DTPA is essential for liver imaging. Compared with dynamic enhanced CT and enhanced MRI of Gd-DTPA, the enhanced MRI of Gd-EOB-DTPA has higher detection sensitivity for the diagnosis of HCC with rich or poor blood supply [15]. Compared with other imaging methods, Gd-EOB-DTPA-enhanced MRI can significantly improve the detection rate and diagnostic accuracy for early liver cancer [26]. Although the feasibility of conventional enhanced MRI in the diagnosis of HCC has been widely recognized, the accuracy and sensitivity for the diagnosis of small HCC with a diameter of < 2.0 cm remains inadequate [27] and fails to fully meet clinical needs. Imbriaco et al. [11] performed the enhanced MRI of Gd-EOB-DTPA on 73 patients and reported that the enhanced MRI of Gd-EOB-DTPA could significantly improve the diagnostic accuracy of lesions with a diameter of < 2.0 cm. The results reported by Wu et al. revealed that the diagnostic sensitivity and specificity of Gd-EOB-DTPA-enhanced MRI for liver cancer with a diameter of < 2.0 cm in patients with chronic liver disease was 0.95 and 0.89 [28], respectively. Kierans et al. reported that the sensitivity and specificity of Gd-EOB-DTPA-enhanced MRI for the diagnosis of liver cancer with a diameter of ≤ 2 cm was 0.92 and 0.95 [14], respectively. A number of studies have revealed that enhanced MRI with Gd-EOB-DTPA is more accurate for diagnosing small HCC (diameter ≤ 2.0 cm), when compared to MSCT multi-phase dynamic enhanced scan, MRI plain scan, and dynamic conventional contrast-enhanced scan [14, 16–18]. Due to its unique advantages [16, 29], Gd-EOB-DTPA significantly improves the detection rate of small liver cancer. Therefore, some scholars have considered that the combination of hepatobiliary stage imaging can improve the accurate diagnosis rate and sensitivity for detecting HCC (≤ 2 cm). The enhancement of Gd-EOB-DTPA can not only reflect the enhancement effect of common contrast agents, but also improve the specific manifestations at the hepatobiliary stage, which can be used as radiographic features of cirrhotic nodules and small HCC.

A related study revealed the atypical arterial enhancement pattern of some early liver cancers, namely, no or mild strengthening [30]. These lesions might be in the process of development from high-grade dysplastic nodules (HGDN) to early HCC. The reason was because the blood supply was not entirely through the artery blood supply in the early stage of liver cancer, with a portal venous blood flow decrease. Hence, the Gd-EOB-DTPA-enhanced MRI lesions would be shown as an atypical enhanced mode. Lesions with organic anion transporting polypeptide 8 (OATP8) expression gradually decreased, and the selective absorption of contrast agents for liver cells also decreased, which shows the hypointensity in the hepatobiliary period. The high-risk nodules, which might progress to rich blood supply lesions, was similar to those in early HCC imaging features, making the differential diagnosis relatively difficult. It has been generally considered that lesion size, the T1WI and T2WI signals, diffusion-weighted imaging (DWI), lipid content, and other relevant information are correlated to the development of HGDN into hemato-rich HCC. The meta-analysis conducted by Li et al. [31] revealed the sensitivity (88%) and specificity (96%) of Gd-EOB-DTPA dynamic enhanced MRI combined with DWI in the diagnosis of HCC. Gd-EOB-DTPA MRI combined with intravoxel incoherent motion DWI (IVIM-DWI) has high clinical value in the effective evaluation of the function and metabolism level of cancerous nodules. However, this has been rarely reported in relevant clinical studies at home and abroad.

Approximately 6–15% of small HCCs exhibited the atypical enhancement mode at the hepatobiliary specific stage [32], such as small liver cancer, liver focal nodule hyperplasia (FNH), and FNH-like nodules in the liver specific period, and all presented with an obvious reinforcement, showing its isointensity or hyperintensity, when compared to normal liver parenchyma. However, it is difficult to distinguish these by means of the conventional imaging method [22]. The Gd-EOB-DTPA-enhanced MRI has a unique advantage in the diagnosis and differentiation of atypical small HCC nodules and FNH-like nodules. A retrospective analysis of 20 patients with atypical nodular HCC and 21 patients with FNH-like nodules suggested that compared to FNH-like nodules, HCC nodules exhibited similar enhancement pattern in the hepatobiliary stage, but had a significant washout effect on the portal venous phase and/or transitional phase (*P* < 0.0001). When this was used in the diagnosis of atypical HCC nodules, the sensitivity was 90% and the specificity reached as high as 100%. The multivariate logistic regression analysis revealed that the washout appearance was the only independent imaging feature associated with HCC (odds ratio, 7.019; *P* = 0.042) [22]. Wang et al. suggested the use of Gd-EOB-DTPA-enhanced MRI instead of traditional Gd-DTPA-enhanced MRI for HCC patients with atypical nodules [32].

Patients always show varying degrees of increase in AFP [33], and a high level of AFP indicates poor prognosis [34]. In some HCC patients with elevated AFP, microtumor lesions cannot be detected by enhanced CT and conventional MRI. In the early stage of small HCC, these lesions presented with isointensity and hypointensity on the T1WI, and slight hyperintensity on the T2WI in conventional MRI, but the enhancement on the arterial phase was not obvious during the multi-phase dynamic enhancement scan. Gd-EOB-DTPA has the capability of demonstrating the characteristic hypointensity of small HCCs on the specific hepatobiliary phase, which is more conducive for the characterization of liver focal lesions, and the detection of small HCCs with a diameter of ≤ 1.0 cm [18, 27, 35]. There are few research reports at home and abroad. Hence, further verification studies are needed. In some chronic liver disease patients, AFP can also increase, and there are also some patients with HCC showing AFP negative [36]. Enhanced CT and conventional enhanced MRI only detect the focal arterial perfusion abnormalities of mild enhanced focus, which cannot be clearly detected in the other sequences. However, if Gd-EOB-DTPA-enhanced MRI is used, which can show the characteristic imaging feature of hypointensity in the hepatobiliary phase, this would enhance the detection rate of small lesions.

#### Clinical application for treatment programs

At present, the treatment methods for small HCC mainly include surgical resection, liver transplantation, and local ablation. In clinic, specific treatment methods are mainly selected according to the size, number, location, and invasiveness of the lesions and liver function of patients [37]. However, after radical treatment, patients with early liver cancer have a high intrahepatic recurrence rate, and the survival rate is still not satisfactory [38]. This may be correlated to the failure to detect metastases before the first radical treatment [6]. The detection results of potentially suspicious small HCC lesions would affect the preoperative BCLC staging of some patients. Some scholars have considered that for liver cancer patients with BCLC stage B and good liver reserve function, such as a number of tumors of ≤ 3, the impact of surgical resection would be better, and there would be no statistically significant difference between the impact of surgical resection and transcatheter arterial chemoembolization treatment in patients with a number of tumors of > 3 [39]. To a large extent, the prognosis of HCC depends on the stage at the time of diagnosis. Hence, the scientific staging and selection of the best and comprehensive treatment plan are the keys to improve the prognosis of HCC patients [40]. Some studies have revealed that based on conventional dynamic enhanced CT, further enhanced MRI examinations with Gd-EOB-DTPA can detect the undetected HCC lesions in patients of BCLC grade 0 or grade A. Gd-EOB-DTPA-enhanced MRI prior to treatment has higher diagnostic accuracy and sensitivity, and can improve the overall survival rate of patients with single HCC, when compared to dynamic enhanced CT examination [6]. The hepatobiliary specific period of Gd-EOB-DTPA-enhanced MRI can detect additional small HCCs with a diameter of 1–2 cm, which is beneficial for the selection of more suitable candidates for liver transplantation [41]. Gd-EOB-DTPA-enhanced MRI can improve the diagnostic accuracy of small HCCs and provide assistance for the formulation of surgical plans.

The HCC associated with hepatitis B-related cirrhosis in the early stage often lack of typical clinical symptoms. When its clinical symptoms become clear, the tumor is often at the middle-late stage, with higher degree of malignancy. Thus, the opportunity for cure is lost, with poor prognosis and higher mortality. Consequently, it is necessary to timely diagnose small liver cancer in the background of hepatitis B-related cirrhosis in clinical practice, in order to allow for its early and timely treatment. The combination of AFP and Gd-EOB-DTPA-enhanced MRI can reflect the differentiation status of HCC cells and may make additional treatment options available for patients with early HCC, who are about to undergo surgery [23].

#### Application for predicting the degree of differentiation, recurrence, and prognosis

Under the Edmondson-Steiner grading system, different degrees of HCC differentiation have been proven to be correlated to the prognosis of HCC [42], suggesting that enhanced MRI of Gd-EOB-DTPA can be used to assess the degree of HCC differentiation and thereby predict the prognosis of HCC patients. Studies have shown that the enhancement degree of Gd-EOB-DTPA-enhanced MRI in hepatobiliary stage lesions is positively correlated with the differentiation degree of HCC to a certain extent. That is, due to the good differentiation degree of highly differentiated HCC, some normal functional hepatocytes can absorb contrast agents and present isointense and hyperintense signals in the hepatobiliary specific stage and vice versa [43]. Lee et al. also reported that highly differentiated HCCs and significant bile secretors presented with isointense and hyperintense signals in the hepatobiliary-specific phase of Gd-EOB-DTPA-enhanced MRI [44]. Qin et al. reported that T1 mapping parameters are helpful for predicting the grading and recurrence of HCC [45], and their results indicated that T1 (L-h)/H (%) is positively correlated with the Edmondson-Steiner grading of HCC. Meanwhile, it was suggested that compared with grade II and III HCC, grade I HCC has the lowest cumulative recurrence rate, and that patients with grade III HCC had a higher recurrence rate, when compared with those with grade II HCC, which was 800 days after resection. Hence, the higher the grade of HCC patients, the higher the recurrence rate. The preoperative grading of HCC by T1 mapping on the Gd-EOB-DTPA-enhanced MRI would be helpful for determining a more appropriate treatment for HCC patients. However, some scholars have investigated the correlation between the signal intensity of hepatobiliary specific stage lesions and the differentiation degree of HCC, and these led to contradictory conclusions. A total of 22 cases with different degrees of differentiation of HCC were retrospectively analyzed by Narita et al. [46]. They found that not all highly differentiated HCC were characterized by liver period high intake, and that patients with liver positive intake, when compared to those with negative intake, were associated with a higher OATP1B3 protein expression. They reached a conclusion that was contrary to previous reports. That is, the enhancement degree of Gd-EOB-DTPA-enhanced MRI hepatobiliary lesion was correlated to the tumor OATP1B3 protein expression level. Asayama et al. also concluded that the uptake of Gd-EOB-DTPA by HCC is not correlated to the degree of tumor differentiation [47]. In general, the relationship between the intensity of the Gd-EOB-DTPA signal at hepatobiliary stage and the degree of differentiation has not been completely cleared, which deserves further study.

The early detection of recurrent lesions after HCC surgery is of great significance for prolonging the survival time of patients. A prospective study used Gd-EOB-DTPA-enhanced MRI to examine 138 cases of BCLC level 0 or grade A and Child-Pugh grade A non-recurrent early HCC patients. Patients with a postoperative recurrence rate and survival rate were analyzed, and the results revealed that the patients with non-rich blood supply nodules and a hypointense signal had significantly higher recurrence rates (*P* < 0.0001), but had significantly lower survival rates (*P* = 0.0108), suggesting poor prognosis [48]. The combination of Gd-EOB-DTPA-enhanced MRI and weighted imaging technology can obtain more qualitative and quantitative data on the degree of HCC differentiation, and the degree of microvascular infiltration or treatment response, thereby providing more data support for the prognosis assessment of HCC patients [49]. The enhanced MRI data analysis of Gd-EOB-DTPA in 61 HCC patients revealed that the uneven boundary of hepatobiliary HCC lesions was associated with portal vein tumor invasion (OR = 18.814, *P* = 0.024) and intrahepatic metastasis (OR = 6.498, *P* = 0.036). The evaluation of portal vein invasion, intrahepatic metastasis, and recurrence in patients within 1 year after hepatectomy revealed that the unsmooth HCC lesions in the hepatobiliary stage of Gd-EOB-DTPA-enhanced MRI were associated with early recurrence after hepatectomy (OR = 4.306, *P* = 0.04) [50]. When liver cells are injured, the ability of liver cells to absorb the gadolinium disodium plug decreases, and the signal detected by MRI weakens. It can be determined whether the liver function is impaired by determining the changes in gadolinium disodium plug acid detected by the Gd-EOB-DTPA-enhanced MRI. The Gd-EOB-DTPA-enhanced MRI has great potential in the prediction of the evaluation of liver function. A study revealed that Gd-EOB-DTPA-enhanced T1 relaxometry, in combination with liver volume, may have the potential to become a novel tool for monitoring liver function [51]. Araki et al. used Gd-EOB-DTPA-enhanced MRI to determine whether functional remnant liver volumetry could predict post-hepatectomy liver failure. The retrospective analysis of 155 cases of hepatectomy revealed that the functional remnant liver volumetry was the most reliable predictor (*P* = 0.013). This result was reliable, in terms of sensitivity (100%), specificity (77.3%), and accuracy (80.8%) [52]. These researches indicate that the application of Gd-EOB-DTPA-enhanced MRI may have an important role in predicting the recurrence of liver cancer and its postoperative prognosis, providing important information for the preoperative and postoperative evaluation of liver cancer patients.

#### Deficiencies and limitations

##### Pharmacoeconomics

With the improvement of diagnostic accuracy, the selection of Gd-EOB-DTPA-enhanced MRI for HCC patients may lead to a higher initial diagnostic cost [3]. However, high-precision diagnosis can reduce additional diagnostic procedures and unnecessary treatment, thereby reducing the overall treatment cost of patients [53]. A cost-benefit analysis in Japan used the Markov model to evaluate the cost-effectiveness of extracellular contrast media-enhanced MRI (ECCM-MRI), enhanced CT, and Gd-EOB-DTPA-enhanced MRI in the diagnosis and treatment of HCC patients. The results revealed that the direct cost of ECCM-MRI, enhanced CT, and Gd-EOB-DTPA-enhanced MRI was 2,365,421 yen, 2,482,608 yen, and 2,174,869 yen, respectively, and the quality-adjusted life year (QALYs) was 9.303 QALYs, 9.215 QALYs, and 9.502 QALYs, respectively. It can be observed that Gd-COB-DTPA-enhanced MRI has the lowest cost and the longest QALYs, with the highest clinical benefits [54]. A health economics study also revealed that the cost of using Gd-EOB-DTPA for enhanced MRI in the diagnosis of suspected HCC patients in South Korea was lower than that of ECCM-MRI and multi-row spiral CT, from the perspective of both patients and hospitals [55]. The results for the health economics in China revealed that the total cost of diagnosis and treatment of patients using Gd-EOB-DTPA-enhanced MRI is similar to that of patients who used multi-row spiral CT, when patients visit a doctor for the first time. However, this would be lower than that of patients who used ECCM-MRI. (Gd-EOB-DTPA-enhanced MRI costs 30,360 yuan vs. 30,803 yuan for multi-row spiral CT vs. 31,465 yuan for ECCM-MRI.) This suggests that Gd-EOB-DTPA-enhanced MRI should be used as the primary imaging modality for HCC and other types of tumors in China [3].

##### Application limitations of Gd-EOB-DTPA

The reinforcement degree of Gd-EOB-DTPA-enhanced MRI for hepatic vessels (artery, portal vein) and the liver parenchyma of HCC were lower than that of Gd-DTPA [56]. The main reason is that the standard dose of Gd-EOB-DTPA is 1/4 of the dose of Gd-DTPA (0.025 and 0.100 mmol/kg, respectively), and the relaxation ratio of 1.5 t MR between the two at 37 °C is 1.7. In practice, the low enhancement of arterial lesions would influence the diagnostic accuracy of the lesions. However, for patients with severe cirrhosis (especially Child-Pugh grade C), the degree of enhancement of liver parenchyma and the time of bile system excretion are correlated to the severity of liver function damage, and the weak and uneven enhancement of liver parenchyma at the specific stage of liver and gallbladder would affect the detection of these lesions. Therefore, for patients with chronic liver disease and/or cirrhosis, the hepatobiliary specific scanning time can be appropriately extended according to the specific situation [57].

## Conclusion

At present, Gd-EOB-DTPA-enhanced MRI has the diagnostic capability for small HCCs with a diameter of ≤ 2 cm. Many scholars continue to explore the diagnostic accuracy and sensitivity of small liver cancer with a diameter of ≤ 1 cm. It has been considered that in the future, Gd-EOB-DTPA-enhanced MRI will have a broader application prospect in the early diagnosis of small liver cancer with a diameter of ≤ 1 cm, thereby providing a more valuable reference for the clinical preoperative evaluation and selection of surgical treatment options. Gd-EOB-DTPA-enhanced MRI also has large research space for the differentiation degree, prediction of recurrence, and prognosis of HCC. Furthermore, the special excretory pathway of Gd-EOB-DTPA will also have a broader development prospect in the diagnosis and research of liver and biliary tract diseases.

## Data Availability

Not applicable.

## Abbreviations

HCC: Hepatocellular carcinoma
HBV: Hepatitis B virus
BCLC: Barcelona Clinic Liver Cancer
CT: Computed tomography
MRI: Magnetic resonance imaging
AFP: Alpha fetoprotein
Gd-EOB-DTPA: Gadolinium ethoxybenzyldiethylenetriaminepentaacetic acid
EOB: Ethoxybenzyl
OATP1B3: Organic anion transporting polypeptide 1B3
MRP2: Multidrug resistance-associated protein 2
LI-RADS: Liver imaging report and data system
HGDN: High-grade dysplastic nodule
OATP8: Organic anion transporting polypeptide 8
DWI: Diffusion-weighted imaging
IVIM-DWI: Intravoxel incoherent motion DWI
FNH: Focal nodule hyperplasia
